# Osteoporosis management within UK care homes: a modified Delphi consensus

**DOI:** 10.3399/BJGPO.2024.0268

**Published:** 2025-12-19

**Authors:** Sunil Nedungayil, Shelley Douglas, Jill Griffin, Lewis Sutherland, Tahir Masud

**Affiliations:** 1 Musculoskeletal, Pain and Rheumatology Services,The Castle Medical Group and East Lancashire Hospital, NHS Trust, Blackburn, UK; 2 Burnley-West Primary Care Network, Lancashire and South Cumbria Integrated Care Board, Burnley, UK; 3 Clinical Engagement, Royal Osteoporosis Society, Bath, UK; 4 North Tyneside General Hospital, North Shields, Tyne and Wear, UK; 5 Geriatric and General Medicine, Nottingham University Hospitals NHS Trust - Department of Healthcare for Older People, Nottingham, UK

**Keywords:** osteoporosis, aged, Delphi technique, primary healthcare, general practitioners, fragility fracture

## Abstract

**Background:**

Osteoporosis is a chronic disease characterised by decreased bone mineral density (BMD) and increased fracture risk. Osteoporosis disproportionately affects residential care home populations.

**Aim:**

To develop recommendations aimed at improving osteoporosis management in UK care homes.

**Design & setting:**

Modified Delphi study of UK geriatric and osteoporosis care.

**Method:**

A steering group of six UK experts in geriatric medicine and osteoporosis care convened to discuss challenges in osteoporosis management within care homes. Forty-five consensus statements were developed and tested in a survey distributed to their peers (targeting 150 responses). Responders rated their agreement on a four-point Likert scale. Consensus was defined *a priori* as ≥75% strong agreement or very strong agreement as ≥90%. The group reconvened to analyse the results and generate recommendations.

**Results:**

In total, 101 survey responses were received from clinicians and care home managers representing all UK regions; 39.6% of responders had >20 years’ experience in their role. Consensus was achieved for most (42/45 [93.3%]) statements and very high agreement achieved for two-thirds (66.7%). Therefore, the survey window was not extended to meet the response target. Nine recommendations to improve osteoporosis and bone health care across the UK primary care network were developed. These emphasise the need for coordinated referrals, treatment plans, and bone health education across primary and secondary care, including care homes. A potential care pathway was generated incorporating these recommendations.

**Conclusion:**

Our study highlights gaps in osteoporosis care in UK care homes, including communication issues. A proactive approach to bone health is encouraged to improve patient outcomes and help alleviate the burden osteoporosis presents.

## How this fits in

Osteoporosis disproportionately affects residential care home residents. However, guidelines specific to the management of osteoporosis in care home residents are lacking. Using the modified Delphi method, this study proposes best-practice guidance for osteoporosis management within the UK care home network.

## Introduction

Osteoporosis is a chronic skeletal disease that reduces bone mineral density (BMD) and increases fracture risk.^1^ It is prevalent in older individuals and post-menopausal women.^2^ In the UK, approximately one in two women and one in five men aged >50 years will experience one or more fragility fractures (sustained from a fall from standing height or less) in their lifetime.^3,4^ Approximately, 519 000–549 000 fragility fractures occur in the UK annually, which is expected to increase 20%–23% by 2030.^5^ Prescribing data for GPs shows only one-third of individuals are receiving anti-osteoporosis medication 1 year after experiencing a fragility fracture.^5^

While typically asymptomatic at onset, osteoporosis can progress to a debilitating condition. Vertebral and non-vertebral fractures cause pain, reduce quality of life, and impact the ability to undertake daily tasks.^3^ Hip fractures limit mobility and lead to permanent disability in 50% of individuals.^6^ Moreover, 10%–20% of patients are placed in a care home in the year following a hip fracture.^3^ Osteoporosis also has a high cost burden. Pre-COVID-19 pandemic, fragility fractures accounted for 2.4% of NHS spending (approximately £5.4 billion a year),^5^ far exceeding previous cost estimates of £2.2 billion per year by 2020.^7^

Care home residents (CHRs) are three times more likely to fall than the general population, and 10 times more likely to sustain significant injuries requiring hospitalisation as a result.^8^ This may be because CHRs tend to be frailer with more complex medical needs.^9^ However, the NHS reports 35%–40% of emergency hospital admissions from care homes are avoidable,^10^ suggesting the high rates of injury and hospitalisation in CHRs may be owing to suboptimal osteoporosis care.^5,9,11^ Indeed, Gerasimaviciute *et al* reported only 16.7% of CHRs received osteoporosis treatment post-fracture, with fewer than half (45.9%) continuing treatment for 12 months and only one-quarter continuing treatment after 24 months.^9^

Despite publication of recognised guidelines, real-world evidence suggests little improvement in the care of high-risk CHRs compared with older community residents.^9^ Moreover, guidelines specific to CHRs are lacking. We conducted a Delphi study to build consensus around an optimal care model for fracture and osteoporosis assessment and management within UK care homes.

## Method

This study was facilitated by an organisation independent of the sponsoring company, with study participants blinded to the sponsoring organisation.

In October 2022 a study protocol was developed and registered following the standard processes of the funding agent. A context review of the literature, focusing on the past 5 years, was undertaken by an independent Delphi facilitator. PubMed, Google Scholar, and Embase were searched, with articles screened and used to develop a briefing document outlining the project scope, which was disseminated to potential steering group (SG) members.

The modified Delphi process is outlined in Supplementary Figure S1. A SG of six UK experts in geriatric medicine and osteoporosis care were contacted via email by the independent facilitator and convened virtually in January 2023. The SG comprised a care home manager (CHM), a care commissioner, a representative of the Royal Osteoporosis Society (ROS), a consultant in geriatric medicine, a GP specialising in musculoskeletal disease, and a rheumatology pharmacist. Individuals were chosen based on their current role, publication history, and background engaging in best-practice development.

During the first meeting, the SG discussed the current challenges surrounding fracture risk and osteoporosis management within care homes. From this discussion, the following four domains of focus were identified:

1. Identification, assessment of risk factors, and stratification;
2. Fracture prevention (management of osteoporosis and falls prevention);
3. Cross-functional alignment and collaboration (including transfer of care and commissioning expectations)
4. Education.

The SG discussed these domains in detail and collaborated with the study facilitator to develop an initial set of 47 statements. The SG independently reviewed these statements to determine their validity and, where necessary, proposed edits. The independent facilitator then developed a final set of statements, which was ratified by the SG and used to generate a survey for wider testing.

The SG distributed the survey using a convenience sampling approach to members of their UK professional networks via Microsoft Forms. Inclusion criteria stated all responders had to be involved in the provision of care in care homes and/or fracture and osteoporosis management. Stopping criteria for this phase of testing were: a target of 150 responses (with no specific response rate sought), a 3-month survey window, and 90% of statements passing the threshold for consensus (to establish a stable baseline). A response target of 150 was chosen to provide a comprehensive reflection of the study population. No incentives were provided to responders. The consensus threshold was set at 75%, with ‘strong’ agreement defined as ≥75% but <90%, and ‘very strong’ agreement defined as ≥90%.^12^

The survey captured basic demographic data of responders (region, specialty, and length of time in role) and presented each statement with a four-point Likert scale (‘strongly disagree’, ‘tend to disagree’, ‘tend to agree’, and ‘strongly agree’). Responses were mandatory for all questions. Completion of the survey was voluntary, and consent was implied by participation. The identity of individual responses were anonymised.

Completed surveys were collated and an arithmetic agreement score for each statement calculated. This information was reviewed by the SG in a second meeting (July 2023). Following agreement that the stopping criteria were sufficiently fulfilled, and owing to the strength and stability of agreement seen, further survey rounds were deemed unnecessary. The SG used the statements to develop a series of recommendations, supported by the agreement levels. These recommendations were then used to develop a care pathway. All outputs were independently reviewed and ratified by the SG.

## Results

Of the initial 47 statements created, 12 were retained, 32 were modified, three were removed, and one new statement was added. The ratified set of 45 statements were distributed as the survey.

### Survey responses

**Figure 1.**
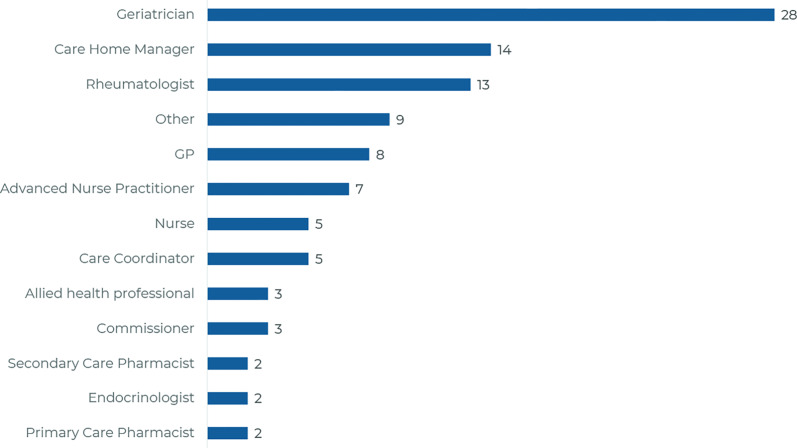
Distribution of responders by role

**Figure 2.**
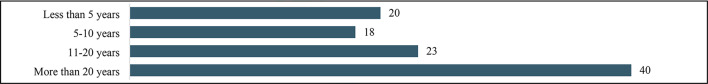
Distribution of responders by experience

Overall, 101 survey responses were received and analysed between April and June 2023. Responders represented a variety of roles (Figure 1). Most (62.4%) reported ≥10 years’ experience in their role; 39.6% reported ≥20 years of experience (Figure 2). Of all UK regions surveyed, Northeast England (Supplementary Figure S2; 26.7%) showed the highest representation. When reviewing these data, the SG agreed it constituted a representative sample.

### Consensus levels

**Figure 3.**
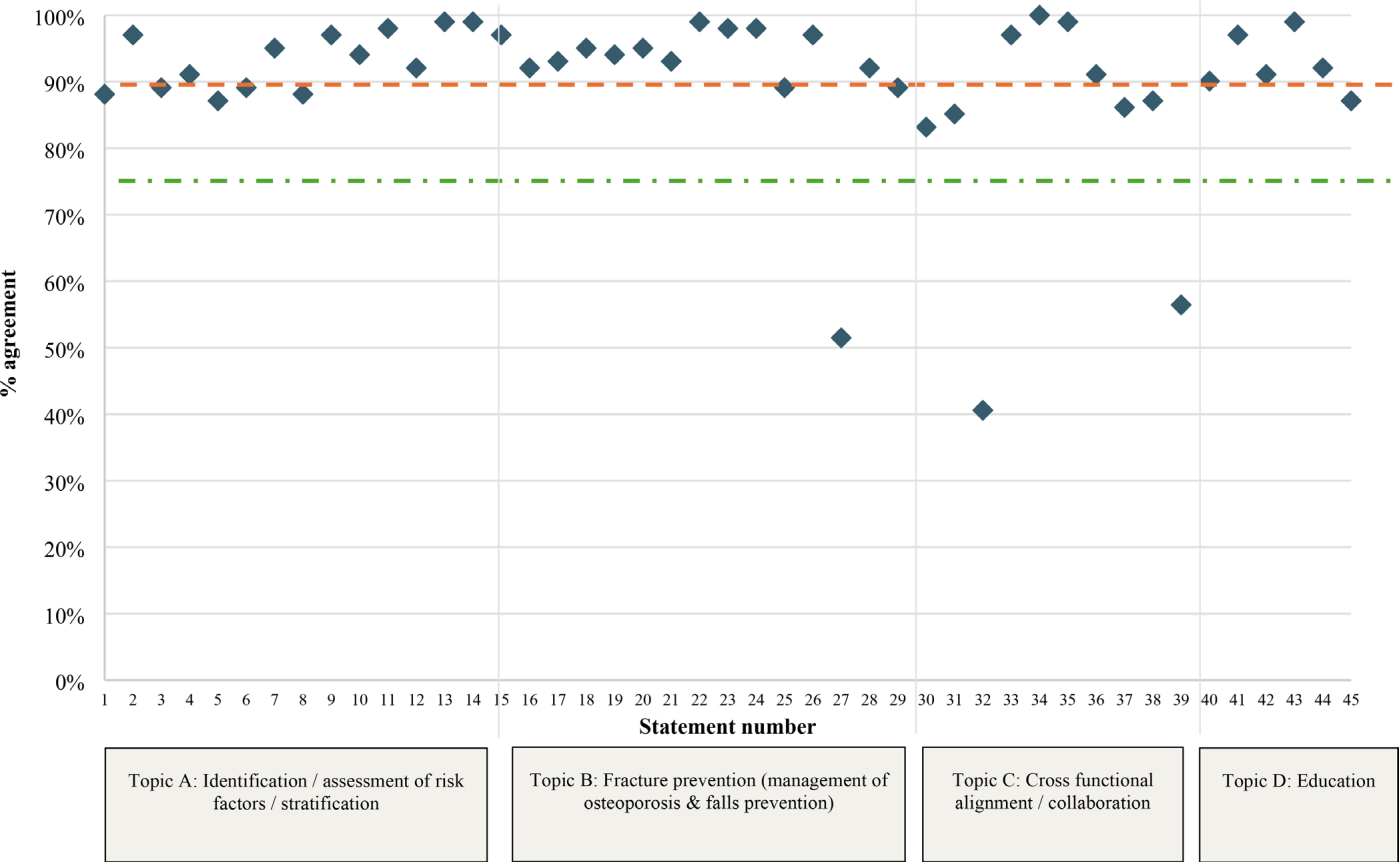
Overall consensus agreement levels by statement. Green line (dot and dash) indicates the ≥75% threshold for strong agreement, orange line (dash) represents the ≥90% threshold for very strong agreement

Survey results are shown in Figure 3 and Supplementary Table S1. Most (*n* = 30, 66.7%) statements achieved very strong agreement (≥90%); three (6.7%) did not reach the threshold (≥75%) for consensus. Overall, consensus was achieved for 42 out of 45 statements (93.3%). Statements with greater than 10% variation from the mean agreement score for at least one role are summarised in Supplementary Table S2. There was no notable variation by geography or time in role (Supplementary Figures S3 and S4). The Likert scale distribution for all statements is presented in Supplementary Figure S5.

### Recommendations to improve osteoporosis care across the UK primary care network

**Figure 4.**
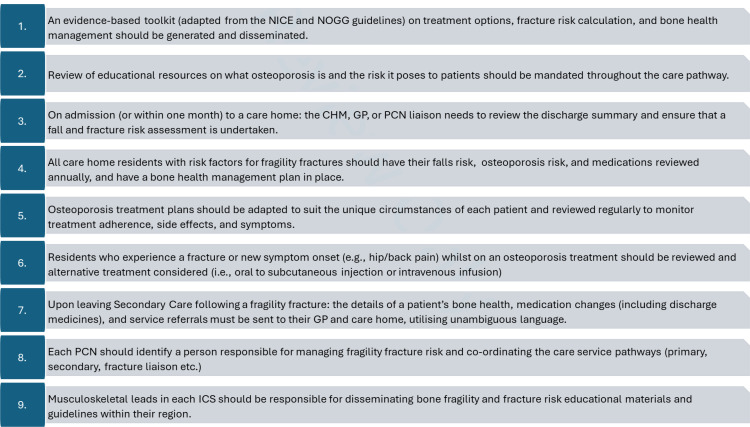
Recommendations to improve osteoporosis health care across the UK primary care network. CHM = care home manager. ICS = integrated care system. NICE = National Institute for Health and Care Excellence. NOGG = National Osteoporosis Guidelines Group. PCN = primary care network

Based on the agreement levels achieved, the SG developed the recommendations shown in Figure 4 and summarised below.

#### Education and guidance

Our first recommendation is to develop an evidence-based and actionable toolkit for bone health, for dissemination across primary care networks (PCNs). This should include guidance on fracture risk calculation, and osteoporosis treatments and dosing regimens. Considering the high to very high agreement observed for statements 12, 14, 15, 17, 19, and 25, we recommend adapting current guidelines from the National Osteoporosis Guideline Group (NOGG)^13–15^ and amalgamating these with National Institute for Health and Care Excellence (NICE) guidelines to produce a single set of standards. The REDUCE hip fracture service implementation toolkit could also be included in our proposed toolkit.^16^ Furthermore, based on the high to very high agreement observed for statements 40–45, we recommend developing resources that promote optimal osteoporosis management across the full care pathway, including care homes (recommendation 2).

#### Risk assessment and prevention

Based on the very high agreement for statement 4, we recommend CHRs have a comprehensive bone health assessment within 1 week of admission to the care home (recommendation 3) and all CHRs have their falls risk, fracture risk, and medications reviewed annually (recommendation 4). This reflects the high consensus that all CHRs should be considered at high risk of falls and other long-term conditions (for example, cardiovascular, respiratory; statement 1) and undergo continuous medical reviews that consider deprescribing medications that increase falls risk, if safe for the patient (statements 12 and 14).

We also recommend treatment plans are tailored to the individual patients’ circumstances (recommendation 5) and reviewed if they experience a fracture or new symptoms (recommendation 6). These recommendations are supported by the high to very high agreement observed for statements 22–26 and 28. In addition, while dual-energy x-ray absorptiometry (DXA) scans are an accepted method for assessing BMD,^17^ they are not practical or suitable for all CHRs and other investigations, recommended by NOGG, may be more appropriate. For example, testing for low levels of serum calcium and vitamin D,^18^ which are associated with an increased risk of falls and fractures,^19,20^ could promote preventive measures of increasing dietary intake of calcium and vitamin D in CHRs awaiting further investigation (for example, FRAX or QFracture risk assessments). This is supported by the very high agreement observed for statement 28, that is, CHRs should have their nutrition optimised for bone health.

#### Commissioning and communication

Very high agreement was reached that communication is poor between primary and secondary care and discharge, with referral letters lacking clarity (statements 39, 33, 34). To address this, we recommend details of a patient’s bone health, medications, and service referrals are sent to the GP and care home following discharge from secondary care for a fragility fracture (recommendation 7).

Agreement was not reached that there are clear guidelines for transferring patients into care homes and care homes are aware of their main contact in the PCN (statements 32 and 39). To address this gap, we recommend each PCN identifies a dedicated bone health coordinator (recommendation 8), and each integrated care board (ICB) has a nominated musculoskeletal (MSK) lead (recommendation 9).

Of note, while a proportion of CHRs often would benefit from zoledronic acid infusions, patients typically have to attend secondary care services to receive their infusions. Some areas of the UK have successfully implemented services to allow administration of intravenous (IV) treatments in the community.^21,22^ Such services could enable administration of IV zoledronic acid in care homes and negate the need to transfer residents to hospital. Therefore, we suggest other osteoporosis services consider this approach.

## Discussion

### Summary

Osteoporosis places a high burden on residential care homes, with improvements in osteoporosis management urgently needed. Our modified Delphi method solicited the opinions of healthcare professionals involved in care home provision and osteoporosis management, which highlight care gaps and the need for improved multidisciplinary collaboration across primary and secondary care. Based on the consensus of these healthcare professionals, we provide recommendations for osteoporosis management within the UK care network.

### Strengths and limitations

The study’s key strengths are the use of consensus levels to develop our recommendations and the high level of consensus achieved across different disciplines, including clinicians, CHMs, and care commissioners. However, the target of 150 responders was not met and the survey was limited to the SG’s network, potentially introducing participant selection bias. To mitigate this, all data were anonymous, the study was executed by a third-party facilitator, and participants were blinded to the sponsor company. Furthermore, the high level of consensus and experience across the responders suggest the data are valid.

The survey used a four-point Likert scale with no 'neither agree nor disagree' option. Using such a scale can lead to middle-option bias, where responses mainly select the 'tend to agree or disagree' categories. However, the majority of responses in our study were positive. While this could have been the result of acquiescence or social agreeability biases, some statements did not reach consensus, and variations between responder subgroups were observed, suggesting this was not the case.

### Comparison with existing literature

Real-world data on osteoporosis management in care homes are currently lacking. In addition, the high or very high consensus for statements 40–45 indicate educational resources promoting management of osteoporosis in care homes were lacking at the time of our study.

Our proposals are consistent with the 'World Falls Guidelines'.^23^ Published after our study, these include a section on reducing falls in care homes, which could inform training of healthcare professionals and care home staff on falls prevention. The very high agreement that discharge letters should use clear and unambiguous language (statements 33 and 34) reflects previous studies that have reported lack of clarity in discharge and referral letters, describing them as *'complex and poor'*.^24^ In addition, difficulties in cross-functional alignment and communication have been reported as potential risks to the success of PCNs.^25^

### Implications for research and practice

**Figure 5.**
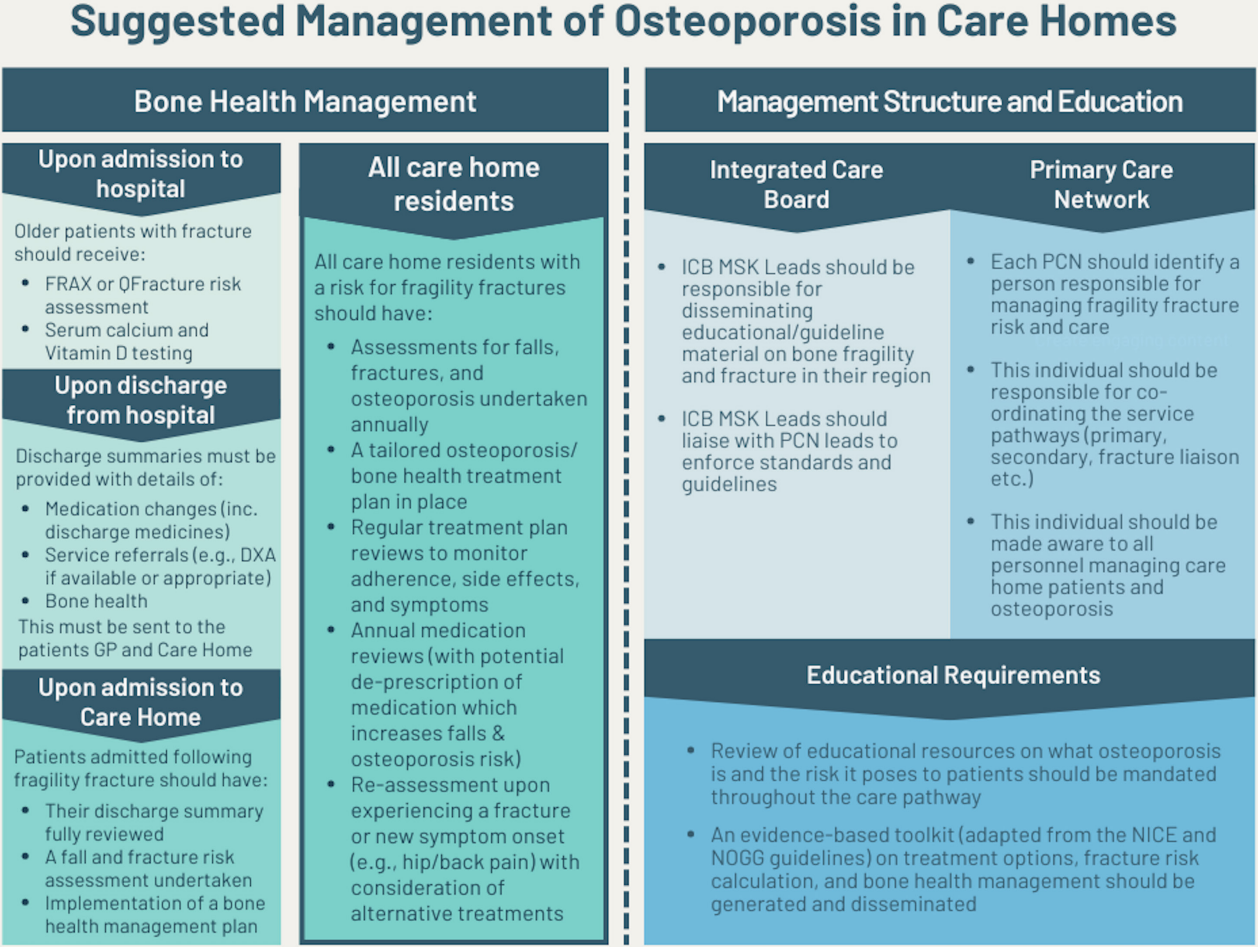
Best-practice guidelines for the management of osteoporosis in care homes. DXA = dual-energy x-ray absorptiometry. FRAX = fracture risk assessment tool. ICB = integrated care board. ICS = integrated care system. MSK = Musculoskeletal. NICE = National Institute for Health and Care Excellence. NOGG = National Osteoporosis Guidelines Group; PCN = primary care network. QFracture = a risk prediction algorithm for osteoporotic fractures

**Figure 6.**
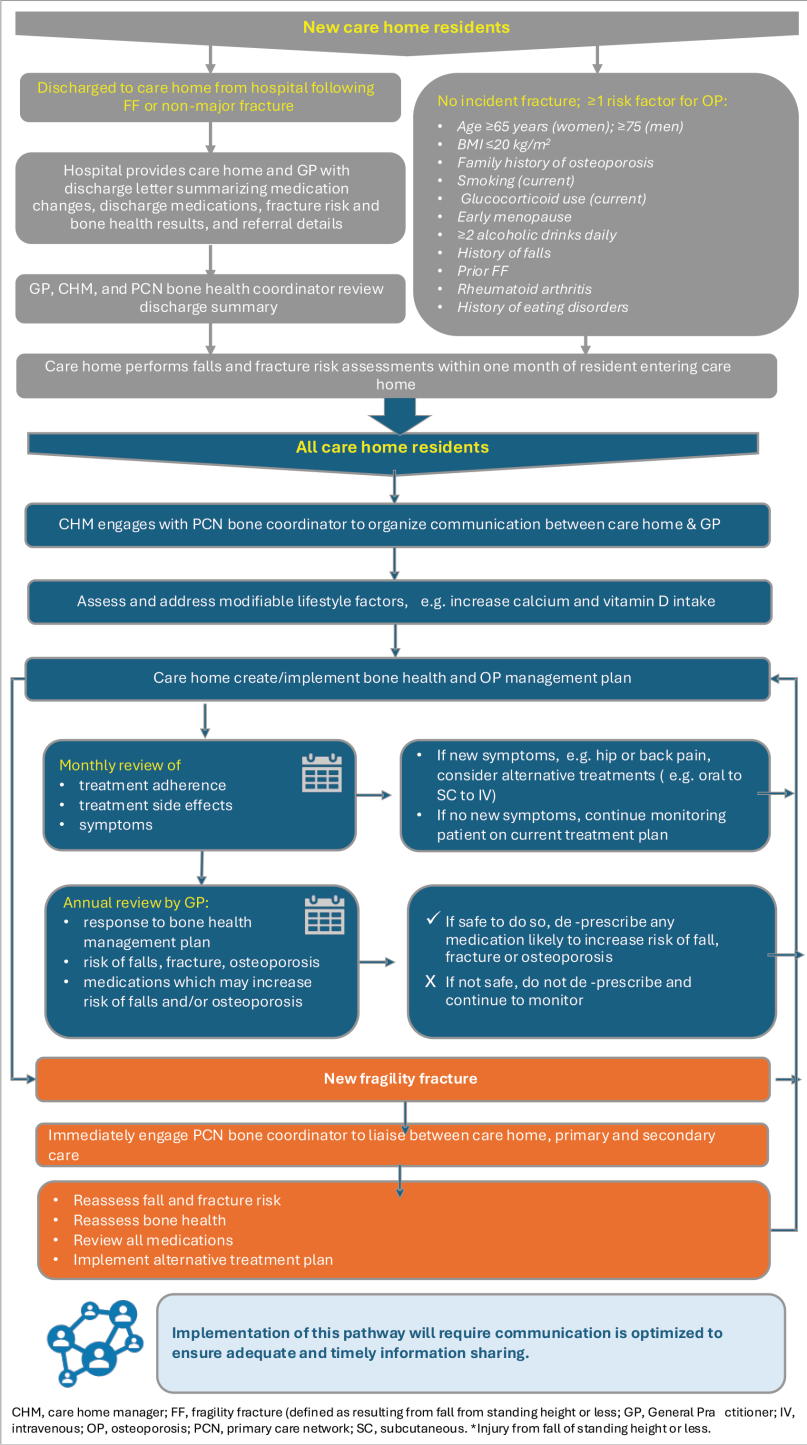
Potential care pathway for management of osteoporosis in care homes. There should be a primary care network (PCN) directed enhanced service (DES) requirement for each PCN to have a process for managing fragility fracture risk and coordinating the care service pathways similar to the one proposed. BMI = body mass index. CHM = care home manager. FF = fragility fracture. IV = intravenous. OP = osteoporosis. PCN = primary care network. SC = subcutaneous

From our recommendations, we developed best-practice guidelines for the management of osteoporosis in care homes (Figure 5) and a suggested care pathway (Figure 6). Collectively these provide a framework for a consistent approach to care and better decision making, and outline responsibilities for clinicians and care leads.

Successful implementation of a multifactorial and adaptable approach, as recommended here, could reduce falls by 40%–50%, which would in turn impact fracture prevalence,^14,15,23^ and reduce the burden fragility fractures place on the NHS. For example, with no universally accepted case-finding strategy for osteoporosis,^13^ and no NHS directives regarding identification of osteoporosis, bone health assessments within 1 week of admission to a care home could be a first step in screening CHRs for osteoporosis. In addition, regular bone health reviews in CHRs could lead to the deprescribing of medications known to increase falls risk, improve adherence to osteoporosis treatments, and reduce the risk of subsequent falls, fractures, and hospitalisations.^26^ More generally, medical reviews are known to balance health and risk^25^ and are recommended in NOGG and NHS guidance.^18,27^

Our recommendations could also improve communication between primary and secondary care. For example, a single contact point for bone health within each PCN would ensure referrals are followed up promptly, a dedicated integrated care system (ICS) lead would ensure guidance on osteoporosis care is enforced, and sending full details of an individual’s bone health, medications, and service referrals to GPs and care homes could increase cross-functional collaboration.

## Data

All pertinent data are presented in the manuscript and supplementary information, so no online dataset is needed. A raw data file can be provided upon reasonable request. Qualified researchers may request data from Amgen clinical studies. Further details are available at Amgen’s website: https://wwwext.amgen.com/science/clinical-trials/clinical-data-transparency-practices/clinical-trial-data-sharing-request
